# Real world evidence for altered communication patterns in individuals with autism spectrum disorder

**DOI:** 10.1038/s41746-025-01545-x

**Published:** 2025-03-11

**Authors:** Mehran Turna, Johannes Eckert, Kristina Meier-Böke, Mamaka Narava, Irini Chaliani, Simon B. Eickhoff, Leonhard Schilbach, Juergen Dukart

**Affiliations:** 1 Research Centre Jülich, Institute of Neuroscience and Medicine, Brain and Behaviour (INM-7), Jülich, Germany; 2 Institute of Systems Neuroscience, Medical Faculty and University Hospital Düsseldorf, Heinrich Heine University Düsseldorf, Düsseldorf, Germany; 3 Department of General Psychiatry 2, LVR-Klinikum Düsseldorf – Kliniken der Heinrich-Heine-Universität Düsseldorf, Düsseldorf, Germany; 4 Medical Faculty, Ludwig-Maximilians-Universität München, München, Germany

**Keywords:** Diagnostic markers, Autism spectrum disorders

## Abstract

Adults with autism spectrum disorder (ASD) may compensate for their social difficulties by resorting to more sequential forms of communication. Here, we study communication preferences in individuals with ASD and neurotypical controls by monitoring smartphone-based communication for verbal, written, and mixed app categories over a period of four months. We find ASD participants to prefer written over verbal communication, underscoring the importance of considering these preferences to facilitate social integration.

Autism Spectrum Disorder (ASD) is defined as a complex neurodevelopmental condition^1^. Although individual experiences with ASD vary, the core diagnostic criteria encompass impaired social interaction and communication as well as the presence of repetitive and/or restricted behaviors^2–4^. Notably, challenges in social communication span issues in language development and lead to social anxiety as well as social isolation. This might be contributing to a distinct communication style among those affected^5,6^. More specifically, clinical insight suggests that individuals with ASD may compensate for their difficulties in intuitively navigating complex face-to-face encounters by resorting to more explicit and sequential formats of communication, which are more in line with the perceptual and communicative abilities of persons with ASD. Similarly, in the interpersonal distance theory of autism, atypical regulation of interpersonal distance arising from distinct neural processing may contribute to discomfort in social interactions. Written communication may serve as an adaptive strategy to mitigate these challenges by allowing greater control over social complexity and interpersonal distance^7^.

From a clinical perspective, there is a critical need for the development of effective social interaction strategies that align with the communication preferences of ASD. Recent advancements in digital communication technologies, such as email and chatbots, have emerged as preferred interaction modalities for individuals with ASD, offering advantages in self-expression and reducing social anxiety^5,8^. This preference for text-based communication over traditional face-to-face or telephonic interactions is supported by literature highlighting the potential of written communication to enhance self-consciousness and self-expression in autistic individuals^9–11^. This exploration of mostly self-reported communication disparities between autistic and neurotypical individuals has indicated a preference for text-based communication modes among those on the autism spectrum, emphasizing their role in healthcare interactions and mitigating social isolation^9,12,13^.

However, all of this previous research is subject to several methodological limitations, relying either on clinical observations or self- and caregiver reporting^5^. These methods are susceptible to different biases such as restrictions to artificial settings and short observational periods for clinical evaluations, both limiting the ecological validity. Furthermore, self- and caregiver reporting are dependent on the ability to correctly recollect information. This could be restricted in individuals with ASD as well as the level of self- and other-awareness^14,15^. The emerging field of digital biomarkers (DB) based on smartphone technologies can address these limitations by directly monitoring daily real-life behavior using participants’ own smartphones. Such DB have been previously successfully leveraged in analyzing gaze patterns, head movements, facial expressions, and motor behaviors to enhance symptom quantification, early detection, and diagnosis of ASD^15–19^. Such observational approaches also allow for direct and more nuanced monitoring of daily life communication behavior and may therefore provide ecologically valid insights into communication preferences in ASD.

Here, we assessed real-life daily communication preferences in adults with ASD by leveraging objective smartphone-based passive data collection. By comparing verbal, written, mixed, and total communication time between ASD and TD, we find individuals with ASD to show a distinct preference for written over verbal communication, which aligns well with previous studies based on self-reports^5,9^.

On average, participants in both groups spent about 27 min with smartphone-based communication (Table 1). About 80% and 66% of this communication occurred in apps with mixed communication possibilities in the ASD and TD groups, respectively.

**Table 1:** Demographic characteristics and statistical description

	ASD	TD	Statistics (Test value (df), *p*-value)
**Age (Mean** **±** **SD)**	34.5 ± 13.1	33.8 ± 14.5	*t*(57.4) = 0.21, *p* = 0.834
***N***	27	33	-
**Gender (F/M)**	8/19	12/21	*χ*²(0.3), *p* = 0.582
**IQ (mean** **±** **SD)**	109 ± 14.9	112 ± 16.6	*t*(57.3) = −0.75, *p* = 0.459
**AQ (mean** **±** **SD)**	39.2 ± 7.3	13 ± 5.1	*t*(43.7) = 15.0, *p* < 0.001
**Verbal communication** (mean ± SD, median[5-95th percentile])	3.1 ± 1.9, 2.6[0.9–6.9]	8.2 ± 2.8, 8.1[3.8–12.8]	-
**Written communication** (mean ± SD, median[5-95th percentile])	2.6 ± 1.1, 2.5[1.3–4.3]	1.1 ± 0.6, 1.0[0.3–2.2]	-
**Mixed communication** (mean ± SD, median[5-95th percentile])	21.5 ± 6.2, 21.0[13.2–32.7]	18.3 ± 4.05, 18.4[12.4–24.2]	-
**Total communication** (mean ± SD, median[5-95th percentile])	27.3 ± 7.1, 27.3[17.3–38.2]	27.6 ± 5.5, 27.8[20.0–35.7]	-

*AQ* Autism Quotient, *IQ* Intelligence Quotient, *SD* standard deviation, *df* degrees of freedom. T-tests were used to test for differences in age, IQ, and AQ. Chi-squared tests were used to compare the gender distribution across groups.

Adults with ASD displayed significantly reduced verbal communication time (*p* = 0.042, *d* = −0.36), but spent significantly more time with written communication (*p* = 0.025, *d* = 0.46) as compared to neurotypical controls. On average, time spent with verbal communication was about 2.6 times lower and the one spent with written communication was about 2.3 times higher in the ASD population. These differences were highly consistent over the study duration, with the ASD group displaying higher written communication on 120 out of 122 days and lower verbal communication on 116 out of 122 days (both *p* < 0.001) (Fig. 1, Supplementary Fig. 1). Time spent with total (*p* = 0.869) and mixed (*p* = 0.394) communication did not differ between the groups. Age, sex, and IQ did not contribute significantly to communication preferences. Communication time did not correlate with the autism quotient (AQ) (mixed communication: *r* = −0.21, *p* = 0.291; total communication: *r* = −0.27, *p* = 0.176; verbal communication: *r* = −0.22, *p* = 0.264; written communication: *r* = −0.09, *p* = 0.658). More descriptive statistics are provided in Table 1.

**Fig. 1:**
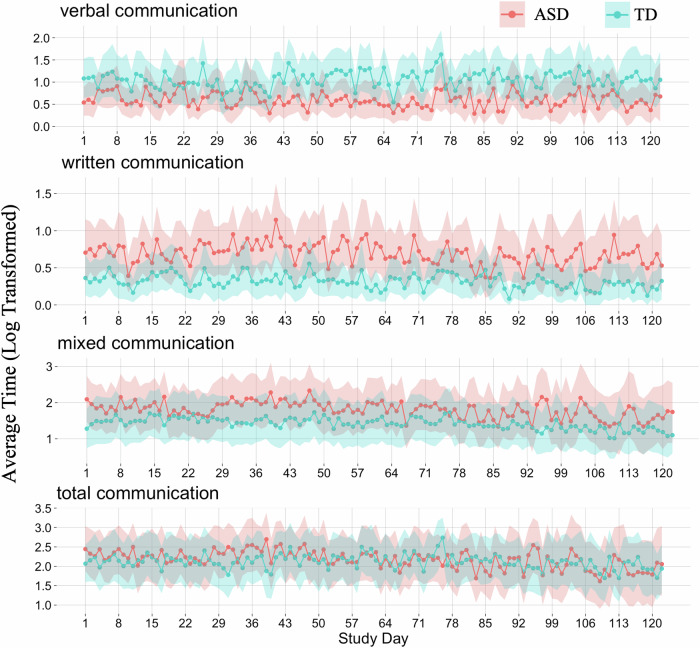
Average daily time spent on smartphones in different categories of communication, compared between Autism Spectrum Disorder (ASD) and typical neurodevelopment (TD) groups. Data was log-transformed. Displayed are the mean and the bootstrapped 95% confidence interval for each study day.

The observed preference for written communication modalities, such as emails and messaging apps, by participants with ASD is in line with their proposed function to reduce social anxiety and enhance self-expression^5,9,10^. This interpretation is also supported by the consistently reduced verbal communication, which was previously linked to increased social anxiety in ASD^20^. Our findings are also in line with the interpersonal distance theory of autism as the preference for written over verbal communication may be due to atypical regulation of interpersonal space, allowing ASD individuals to better manage social complexity and distance^7^.

Although the correlations between communication modalities and AQ scores were statistically non-significant, the observed trends suggest potential patterns in how individuals with ASD use communication technologies based on their AQ scores. This highlights the necessity for more detailed assessments that can capture the subtle nuances of communication preferences. Additionally, employing larger sample sizes or conducting in-depth daily behavioral surveys could improve our understanding and lead to more definitive conclusions.

Considering that we did not collect any information on anxiety this issue should be evaluated in a future study with a specific focus on social anxiety. No significant differences were observed in the use of mixed communication methods (i.e. WhatsApp) or the overall communication time, suggesting that while the mode of communication differs, the overall time spent communicating remains comparable. Although there is a lack of objective demonstration of social engagement in individuals with ASD, previous studies have shown that autistic adults have a desire to form social connections and participate in the community at rates comparable to their neurotypical peers^21–23^. Our findings support this notion, indicating that individuals with ASD are equally engaged in social interactions through their preferred communication modes. They are also in line with the idea that leveraging text-based communication channels and social platforms may have a strong potential to facilitate social engagement in individuals with ASD^11^. Healthcare providers, educators, and caregivers should incorporate written communication methods, such as emails, text messages, or chatbots, to further facilitate communication and reduce social anxiety in ASD^12,13^.

Whilst our findings demonstrate the usefulness of smartphone-based passive data collection to provide ecologically valid quantitative measures of daily communication behaviors, mitigating the biases associated with self-reporting and clinical observations, several limitations warrant consideration when interpreting the observed effects. Besides our cohort’s relatively moderate sample size, the exclusive focus on smartphone-based communication may miss other potentially relevant technologies and may only partially reflect the translation of technology-based communication to actual in-person communication. Another limitation is the omission of iOS users due to data collection constraints imposed by this operating system, which may for example introduce a selection bias. Whilst the categorization of the apps into verbal, written, or mixed was performed blind to the diagnosis and was based on the descriptions in the Google Play Store this approach is inherently limited as there is no exact method to definitively determine the primary usage of an app. Apps used for verbal communication now for example also frequently have chat functionality during the calls. The technically enforced restriction to app usage duration as an indicator of communication preferences may have introduced further biases into our data. It is for example unclear how much of the recorded time was spent on actual communication, as opposed to other activities like re-reading or composing messages, which may not involve direct interaction. This lack of clarity arises from privacy concerns, as direct monitoring of the content and nature of interactions would violate participant confidentiality. Therefore, our findings may not accurately reflect the true extent of active communication and should be confirmed in a future cohort integrating for example also self-reported measures of the specific user interactions.

Lastly, we focused on adults with autism who have a variety of abilities and different support needs across multiple domains. Future studies should also consider a more diverse background as well as explore communication preferences across different age ranges, symptom levels, and consider the impact of various comorbidities.

In summary, we make use of the DB based on smartphone technology advancements to objectively monitor smartphone-based communication preferences in ASD in daily life over a longer period of time. Our findings aim to demonstrate the feasibility of such an observational approach as well as to inform the creation of tailored monitoring, communication, and support strategies, fostering more inclusive and effective support for individuals with ASD.

## Methods

### Study cohort and data collection

We recruited 44 adult individuals with a clinical diagnosis of ASD (the inclusion criteria comprised an IQ of above 70) aside to a control group of 54 individuals with typical neurodevelopment (TD). All participants with ASD received a diagnostic assessment in accordance with the national autism guidelines (Arbeitsgemeinschaft der Wissenschaftlichen Medizinischen Fachgesellschaften (AWMF), 2015), and a formal diagnosis of autism was provided by experienced clinical psychiatrists. Patients were recruited from the “Clinic of Disorders of Social Interaction” at the Department of General Psychiatry 2 at the LVR-Klinikum Düsseldorf.

Passive data collection was conducted using the “JTrack Social” application, which operates passively without necessitating active participation from the participants^24^. This smartphone application continuously collected data over a period of four months, capturing - among other information - the application usage (including timestamps, duration, and names of the applications used). All data was automatically transferred to a secure server at the Research Centre Jülich. Participants were automatically logged out of the study when reaching the study duration. All smartphone-based data was pseudonymized at the source. All potential participants were provided with comprehensive information about the study and informed written consent was obtained from each participant to ensure ethical compliance. All smartphone data was encrypted during transmission using the Hypertext Transfer Protocol Secure (HTTPS). Access to stored data was restricted to authorized personnel, and strict adherence to data security protocols was maintained.

The study was approved by the Ethics Committee of the Medical Faculty of the Heinrich Heine University Düsseldorf, Germany.

### Data Preprocessing and Feature Extraction

Out of 98 recruited participants, all iOS users (*n* = 17) were excluded from further evaluation of communication preferences as iOS technically does not provide information about specific app names making it impossible to estimate the actual communication mode. Furthermore, to ensure sufficient observation duration only users who contributed data for more than half of the study duration (minimum 60 days) were considered (i.e. leaving the study too early or manually stopping data recording) resulting in further *n* = 21 drop-outs. These drop-outs were proportionally equally distributed across ASD and TD groups resulting in *n* = 60 (27 ASD, 33 TD) participants included in further evaluation (Table 1). For these users, the relevant communication applications were categorized based on their primary communication usage into verbal, written, or mixed communication based on app descriptions available in the Android Play Store. Total communication was computed as a sum of these categories. The application usage for each communication app was calculated by summing the daily foreground time, representing the duration users interacted with the app on their screens. For instance, a phone call was classified as verbal communication, while text messaging or email apps were defined as written communication. Dual-functionality apps like “WhatsApp” fell under mixed communication Supplementary Table 1. As app recording times provided by the operating system are not always precise resulting in potential overestimation of phone usage, apps with more than 6 h per application per day were considered as technical outliers and excluded from the analysis. Subsequently, daily average usage for each category within ASD and TD groups was independently computed.

### Statistical analysis

Statistical analyses were performed in Jamovi (version 2.3, https://www.jamovi.org.). General Linear Models with mixed effects were used to test for differences in communication preferences for each of the categories (written, verbal, mixed, and total) between the ASD and TD groups:

$${Communication}\_{time} \sim 1+{group}+{age}+{sex}+{IQ}+(1|{subject})$$

The dependent variables were log-transformed to approximate a normal distribution before fitting the models. All models were adjusted for age, sex, IQ (fixed effects), and subject (random effect). Considering the exploratory nature of this study, the statistical significance threshold was set at *p* < 0.05 uncorrected. Cohen’s d effect sizes were used to estimate the magnitude of the observed differences across all observations. In addition, we computed the sign tests to test for consistency of the group preferences for the observed significant group communication categories comparing the number of days where mean group communication time was higher in ASD as compared to TD. Additionally, the analysis was expanded to investigate correlations between AQ scores and the duration of app usage across different communication modalities: mixed, total, verbal, and written, using the Pearson correlation method.

## Supplementary information

SUPPLEMENTAL MATERIAL

## Data Availability

All data required to create the figures and perform statistical analysis in this paper will be provided upon request to the corresponding author. The list of applications by their primary usage is provided in Supplementary Table 1, in supplement materials.

## Code availability

The code used in the analysis and data preprocessing will be provided upon request to the corresponding author. The JTrack platform is accessible via https://jdash.inm7.de/.
