# Supplementary material for: Real world evidence for altered communication patterns in individuals with autism spectrum disorder
Source: NPJ Digit Med. 2025 Mar 11;8:155. doi: 10.1038/s41746-025-01545-x (PMC11897150; doi:10.1038/s41746-025-01545-x)
Supplement: Supplementary file 1 — SUPPLEMENTAL MATERIAL [file 41746_2025_1545_MOESM1_ESM.pdf]

Supplement Materials for:

Real world evidence for altered communication patterns in individuals with autism spectrum disorder

Authors: Mehran Turna[1,2*], Johannes Eckert[1,2,3], Kristina Meier-Böke[1,2,3], Mamaka Narava[1,2], Irini Chaliani[3], Simon B. Eickhoff[1,2], Leonhard Schilbach[3,4], Juergen Dukart[1,2]

[1] Research Centre Jülich, Institute of Neuroscience and Medicine, Brain and Behaviour (INM-7), Jülich, Germany
[2] Institute of Systems Neuroscience, Medical Faculty and University Hospital Düsseldorf, Heinrich Heine University Düsseldorf, Düsseldorf, Germany
[3] Department of General Psychiatry 2, LVR-Klinikum Düsseldorf – Kliniken der Heinrich-Heine-Universität Düsseldorf, Düsseldorf, Germany
[4] Medical Faculty, Ludwig-Maximilians-Universität München, München, Germany

*Corresponding author:
Mehran Turna,
m.turna@fz-juelich.de

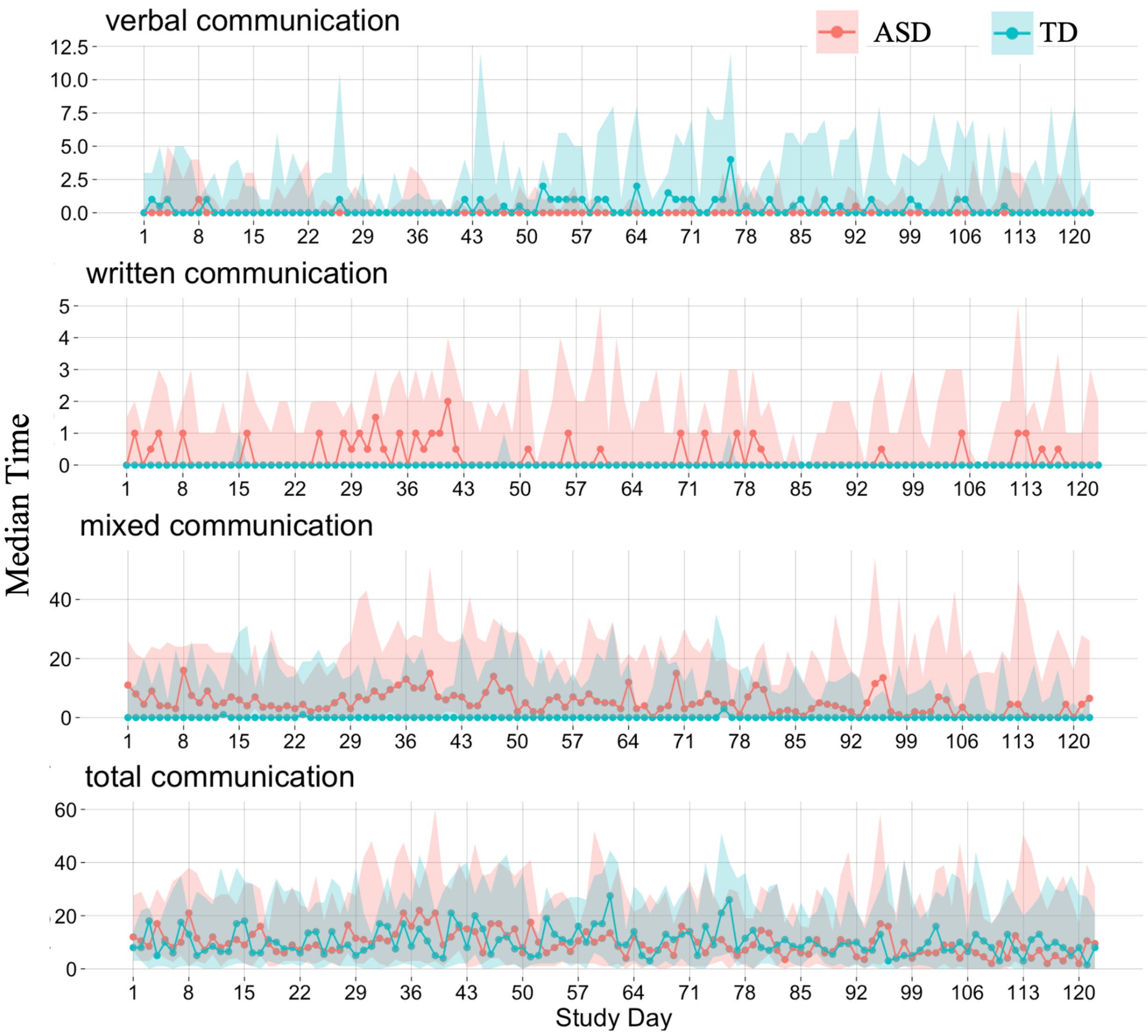

Supplementary Figure 1: Daily Median time spent on different categories of communication applications, compared between Autism Spectrum Disorder (ASD) and typical neurodevelopment (TD) groups. Displayed are the median and the bootstrapped 95% confidence interval for each study day.

Supplementary Table 1: Categorization of applications by their primary usage/purpose

Verbal Communication	Written Communication	Mixed Communication
Skype	Threema	Anrufe/SMS auf anderen Geräten
Teams	Element	Discord
Anruf	Element (Riot.im)	GoToMeeting
Duo	BlueMail	Hangouts
Telefon	Chat	LINE
Telefonanrufe	Currents	Messenger
Voicemail	Yahoo Mail	Messenger Lite
Webex Meet	Dual Messenger	NetSfere
Zoom	E-Mail	Signal
	Freundschaftsdienst	Tandem
	Gmail	Telefonservices
	GMX Mail	Telegram

	K-9 Mail	Viber
	Mail	WhatsApp
	Messages	Wickr Me
	Messaging	Wire
	Outlook	Meet
	Rocket.Chat	KakaoTalk
	Slack	
	SMS Services	
	SMS/MMS	
	Telekom Mail	
	WEB.DE Mail	
